# The Motivational Value Systems Questionnaire (MVSQ): Psychometric Analysis Using a Forced Choice Thurstonian IRT Model

**DOI:** 10.3389/fpsyg.2017.01626

**Published:** 2017-09-20

**Authors:** Josef Merk, Wolff Schlotz, Thomas Falter

**Affiliations:** ^1^Faculty of Business Studies, Regensburg Technical University of Applied Sciences Regensburg, Germany; ^2^Institute of Experimental Psychology, University of Regensburg Regensburg, Germany; ^3^Max Planck Institute for Empirical Aesthetics Frankfurt, Germany

**Keywords:** value systems, assessment, forced-choice format, Thurstonian IRT, empirical reliability, validity

## Abstract

This study presents a new measure of value systems, the Motivational Value Systems Questionnaire (MVSQ), which is based on a theory of value systems by psychologist Clare W. Graves. The purpose of the instrument is to help people identify their personal hierarchies of value systems and thus become more aware of what motivates and demotivates them in work-related contexts. The MVSQ is a forced-choice (FC) measure, making it quicker to complete and more difficult to intentionally distort, but also more difficult to assess its psychometric properties due to ipsativity of FC data compared to rating scales. To overcome limitations of ipsative data, a Thurstonian IRT (TIRT) model was fitted to the questionnaire data, based on a broad sample of *N* = 1,217 professionals and students. Comparison of normative (IRT) scale scores and ipsative scores suggested that MVSQ IRT scores are largely freed from restrictions due to ipsativity and thus allow interindividual comparison of scale scores. Empirical reliability was estimated using a sample-based simulation approach which showed acceptable and good estimates and, on average, slightly higher test-retest reliabilities. Further, validation studies provided evidence on both construct validity and criterion-related validity. Scale score correlations and associations of scores with both age and gender were largely in line with theoretically- and empirically-based expectations, and results of a multitrait-multimethod analysis supports convergent and discriminant construct validity. Criterion validity was assessed by examining the relation of value system preferences to departmental affiliation which revealed significant relations in line with prior hypothesizing. These findings demonstrate the good psychometric properties of the MVSQ and support its application in the assessment of value systems in work-related contexts.

## 1. Introduction

Every day, people take many work-related decisions, some simple and some more complex. To name a few examples: How to get to work? Which tasks to do in which order? How to proceed to solve a problem? Which colleague to ask for advice? Chances are high that different people will come to different answers, even if job and context are identical. Likewise, the motivation to pursue a certain action may vary strongly between individuals, depending on the answer a person gives to the above-mentioned questions.

Value systems play an important role in the emergence of motivation and guidance of behavior. They help people to determine subjectively preferable answers to the introductory posed questions under the assumption that they function as psychological criteria to conclude which decisions and action alternatives are desirable (Rohan, 2000; Hitlin and Piliavin, 2004). In the cognitive process of comparing options and identifying preferences, value systems guide people in determining the subjective value and utility of each alternative and thus formulate priorities. The valence is thereby determined by both the congruence of a stimulus with a value system and the rank of the respective value system in the personal hierarchy (Schwartz, 1996). Generally speaking, value systems function as psychological frames of reference and are applicable to actions, objects, situations, events or even persons (Kluckhohn, 1951; Rokeach, 1973).

The development of a new measure of value systems can be justified by at least three reasons. First, an instrument would help people identify their value systems and thus gain more awareness of their psychological frames of reference. It would lead to a higher level of self-determination, because using language to externalize one's thoughts and beliefs often helps people to become clearer on their positions and thus enables them to determine preferable action alternatives. Besides, knowledge about an individual's value systems hierarchy can be used by job and career counselors to provide useful advice on appropriate training and job paths. It could also be applied in human resources and personnel selection to help maximize the fit of applicants or employees to particular jobs. No validated instrument measuring values is based on the model of Graves (1970) which has had some impact in practical management applications in recent years (e.g., Versnel and Koppenol, 2003, 2005; Beck and Cowan, 2007; Keijser and van der Vat, 2009), but has not been examined scientifically. An investigation into the validity of the theory should be the basis of applying it in practice. Second, existing measures of value systems (e.g., Schwartz Value Survey, Schwartz 1992; Circumplex Scales of Interpersonal Values, Locke 2000) that rely on rating scales are—as we will argue—less suitable to measure value systems than ranking scales. The MVSQ was intentionally designed in a full ranking format (without the possibility of ties; we use the term “forced-choice” for this type of ranking format) which represents the comparative nature of value systems. Third, existing ranking instruments of values (e.g., Rokeach Value Survey, Rokeach 1973; Münster Work Value Measure, Krumm et al. 2013) are restricted due to ipsativity of forced-choice (FC) data when conducting interpersonal comparisons of scale scores. In this study, we apply a new approach to model FC data to overcome such restrictions.

The overall purpose of this study was to assess the instrument's psychometric properties and major aims were (a) to apply the Thurstonian IRT (TIRT) model to the FC response data generated by the MVSQ; (b) to compare results of classical ipsative scoring with TIRT scoring with regards to trait inter-correlations and individual scale score profiles; (c) to estimate the reliability of the TIRT trait score estimates; and (d) to explore the validity of TIRT trait scores analyzing both construct and criterion-related validity.

The MVSQ is based on a theory of value systems developed in the 1960–1970s by Clare Graves (1966, 1970, 1971a,b,c, 1974). According to Graves, value systems are ordered hierarchically, describe different motivational systems and determine what a person considers as desirable (Graves, 1970, 1974). All three points are in line with more recent and widely accepted definitons of value systems (e.g., Kluckhohn, 1951; Rokeach, 1973; Schwartz and Bilsky, 1990; Schwartz, 1992, 1994; Rohan, 2000). Graves' approach differs to other values theories in terms of the number of value systems and partly the content of the latent constructs. The theory posits a model of seven value systems that are summarized in Table 1. Additionally, it contains an eighth value system that is, in contrast to the other seven value systems, not accessible to consciousness and does not differ interindividually (Graves, 1970). Thus, it does not qualify as a system of values but rather as a system of basic needs (cf. Locke and Henne, 1986; Locke, 1991, 1997, on the difference between values and needs). It is described by such needs as survival and physiological stability (Graves, 1970). The MVSQ does not, therefore, feature this category as a latent trait.

**Table 1:** Value system denominations (instrumental and terminal) and descriptive values of seven value systems as measured by the Motivational Value Systems Questionnaire (MVSQ).

**Value system**		**Example values**
**Instrumental**	**Terminal**	
Preserving (PR)	Preservation	Tradition, cohesion, continuity, bonding
Doing (DO)	Power	Pace, decisiveness, openness to conflict, simplicity
Complying (CO)	Assurance	Rules, procedures, duty, obligation
Achieving (AC)	Success	Personal success, rewards, goal-orientation, competition
Harmonizing (HA)	Equality	Harmony, consensus, mutuality, collaboration
Understanding (UN)	Freedom	Intellectuality, complexity, theorism, knowledge
Sustaining (SU)	Sustainability	Social relevance, global issues, social responsibility, holism

As for the denomination of value systems, we opted not to follow Graves' now old-fashioned style to use letters (Graves, 1970). Instead, we list both instrumental and terminal values (cf. Graves, 1970; Rokeach, 1973) and focus on instrumental values as denominators of whole value systems for one major reason: Conventionally, nouns are used to describe single values. In order to easily distinguish single values from value systems (that include several values), we chose the less-common instrumental denominators. Additionally, one can argue that an action-related vocabulary (instrumental values) insinuates the conceptual closeness of value systems to motivation (Locke, 1991; Locke and Latham, 2004) more appropriately than static nouns. The labels chosen were derived from the original descriptions of value systems by Graves (1966, 1970, 1974).

As value systems are accessible to consciousness (Graves, 1970; Locke and Henne, 1986; Schwartz and Bilsky, 1987; Latham and Pinder, 2005), they can be measured by self-report questionnaires (McClelland, 1987). Self-report measures of values often reflect one of two different traditions as indicated by their response format. On one side there are advocates of rating, i.e., single stimulus response formats (e.g., Braithwaite and Law, 1985; Schwartz, 1994). On the other side there are proponents of ranking, i.e., forced-choice (FC) responding (e.g., Rokeach, 1973; Harzing et al., 2009). For conceptual and practical reasons we argue that the forced-choice response format offers several advantages over single stimulus response formats when measuring value systems. Conceptually, the FC format directly operationalizes the comparative nature of values systems (Rokeach, 1973; Kamakura and Mazzon, 1991; Meade, 2004) because value systems are used as evaluative criteria to weigh up alternatives and decide on the valence of a cognitive representation compared to one or several others. In contrast, rating scales require the respondent to estimate the absolute importance of a single value system independent of other value systems which is not stringent when considering that value systems are assumed to be hierarchically ordered. From a practical point of view, rating was clearly preferred over ranking until the development of the Thurstonian IRT approach, as restrictions due to ipsativity in statistical analysis do not apply to single stimulus response formats. With the TIRT modeling being more and more applied to FC measures (e.g., Anguiano-Carrasco et al., 2014; Joubert et al., 2015) we see few arguments in favor of continuing to use rating scales when assessing value systems.

## 2. Materials and methods

### 2.1. Scale development

We aimed to develop a multidimensional FC questionnaire consisting of 20 item stems, each with seven items representing one value system each, as described in Table 1. Value systems were operationalized as an individual's preference judgments aggregated across different work-related situations. Each block (one item stem and seven items) was designed to address a common aspect of work. Table 2 show the mapping of blocks to common situations. The pool of situations was derived by interviewing long-time managers and employees on their typical work activities and was counterchecked with literature resources such as Haslam (2009) and Zedeck (2010). In the end, the blocks covered the following situations: decision making, tasks, goal striving, performance, recognition, work load, collaboration, conflict, and work environment (team and organizational culture). Each situation was used to phrase both desired states or preferred behaviors and undesired states or disfavored behaviors per value system. The original item pool consisted of 24–30 items per value system which were generated by reviewing literature resources such as (Graves, 1966, 1970, 1971a,b,c, 1974; Versnel and Koppenol, 2003, 2005; Beck and Cowan, 2007; Keijser and van der Vat, 2009). Items were revised based on feedback from experts on Graves theory regarding item difficulties, accuracy of wording and degree of attractivity within each block. The resulting questionnaire was completed by colleagues, relatives, and friends who gave feedback regarding comprehensibility. This process resulted in a first operational version of the questionnaire. This version was then used in a sample of 729 participants. A small number of item wordings were then revised based on item difficulties and item-total correlations.

**Table 2:** Mapping of MVSQ blocks and work-related situations.

**Work-related situation**	**Block**	
	**Positive**	**Negative**
Task and goal striving	1	11
	7	19
	9	20
Performance	2	15
Work environment	3	13
Recognition	4	12
		14
Conflict	5	17
	6	
Work load	8	16
Decision making	10	18

*Positive and negatve refer to keying of items within block*.

The final version of the MVSQ has 20 blocks with seven items per block (total of 140 items). The item stems of 10 blocks have positive expressions (such as I love, I like, I prefer, I wish) in order to measure one end of the trait continuum and 10 blocks are formulated negatively (e.g., I hate, I cannot stand, I disapprove of, I dont like) representing the other end. All items within a block are keyed in the same direction because value systems are conceived as comparative constructs, and mixing positive and negative items would require neutral item stems which would impose higher cognitive load on respondents. An example block (with corresponding value systems in parentheses) is:

I love tasks where I…

- Carry on traditions and customs. (**Preserving**)
- Make quick decisions without thinking long about it. (**Doing**)
- Have clear guidance through formal requirements. (**Complying**)
- Am able to solve personally important challenges pragmatically. (**Achieving**)
- Work closely together with others. (**Harmonizing**)
- Develop my own theoretical concepts. (**Understanding**)
- Work on the really important societal challenges. (**Sustaining**)

Respondents are given detailed instructions to, first, read all items, secondly, rank the items according to current approval and thirdly, before proceeding to the next block, review the order and, if desired, change an items rank. Additionally, the questionnaire includes demographic questions, such as nationality, gender, age, education and work experience, as well as job function, branch and management level, if applicable. The MVSQ was implemented within an online interface and ranking of the response options uses an intuitive drag-and-drop procedure. Persons with work or work-alike experience, including both professionals and students, can answer the questionnaire. To highlight the role of value systems as the core of motivation (Locke, 1991), the instrument was termed Motivational Value Systems Questionnaire (MVSQ) for research purposes and my_motivation for consulting practice.

### 2.2. Scoring of forced-choice scales

Due to its forced-choice response format, the MVSQ yields fully ipsative data. Often, responses on forced-choice items are scored in a classical way by summing up the ranks of a trait-indicating response over blocks, resulting in ipsative scores with a number of unfavorable properties. Brown and Maydeu-Olivares (2013) note the following limitations: (1) Ipsative scores reflect the relative strength of a trait within an individual, but provide no normative information on trait standing between individuals. Therefore, score interpretation and selection decisions on the basis of ipsative scores are problematic. (2) Ipsative scores are negatively correlated, because higher scores are necessarily associated with lower scores on another trait; the average correlation among the k scales of an ipsative test is −1/(k−1), leading to distorted construct validity estimates, particularly when the true trait score correlations are expected to be positive. (3) The covariances of scale scores of an ipsative test with any external criterion measure sum to zero. Therefore, covariances are distorted, for example, if mostly positive correlations with external criterion variables are expected. (4) Ipsative scoring leads to inconsistent coding of responses (a response might receive a low score despite high true trait standing if other traits are ranked higher by the respondent), and violation of the fundamental assumption of independence of errors due to dependencies of responses within blocks. Therefore, appropriateness of classical reliability estimators is doubtful and measurement precision remains unknown. With the aim of providing a method for distortion-free scoring of ipsative test data, Brown and Maydeu-Olivares (2011) recently developed an item-response theory model for forced-choice questionnaires on the basis of an appropriate response process model using Thurstones law of comparative judgment.

Lastly, there are other approaches to model forced-choice data (cf. Brown, 2014, for an overview), e.g., the Multi-Unidimensional Pairwise-Preference Model by Stark et al. (2005) or the multidimensional unfolding approach by McCloy et al. (2005). These models were developed for ideal-point items (Brown, 2014). As the MVSQ items were designed following the dominance-model, these alternative approaches are not appropriate to model MVSQ data.

### 2.3. Study design

Several samples of German professionals and students were used to assess reliability and validity of the MVSQ. Tests of empirical reliability were based on a sample of *N* = 1,217 professionals and students. Further, data from this sample were used to calculate intercorrelations between value systems, correlations between age and value systems, and differences between gender to investigate the MVSQ's construct validity. To test temporal stability of MVSQ scores, the test was administered twice to a group of *N* = 72 students with an assessment interval of approximately 15 weeks. Further, we examined convergent validity by means of a multitrait-multimethod matrix using a sample of *N* = 102 students who also completed the Schwartz Value Survey (SVS) (Schwartz, 1992; Glöckner-Rist, 2001). Finally, criterion-related validity was analyzed in a sample of *N* = 402 professionals by comparing mean standings on value systems for three different jobs types (sales, research and development and personnel) with the underlying hypothesis that different value systems are characteristic of different jobs. All participants gave written informed consent to use their data for research purposes.

#### 2.3.1. Sample for empirical reliability and construct validity (scale intercorrelations, gender and age)

The sample comprised *N* = 1,217 individuals (520 women, 42.7%) with a mean age of 33 years (SD = 10.8; range = 18–75). Data for the subgroup of professional participants (*n* = 818, 67.2%) were collected from a range of client projects for consulting and self-development purposes. Student participants (*n* = 399, 32.8%) were recruited at the Regensburg Technical University of Applied Sciences (OTH Regensburg), Germany, and were offered comprehensive feedback on their results.

Overall, the participants' mean duration of work experience was 9.6 years (SD = 10.1) but varied widely with 18.3% reporting work experience between 3 months and 1 year, 23.8% between 1 and 5 years 25.1% between 6 and 15 years and 28.7% more than 15 years. On average, professionals reported more work experience (*M* = 13.3 years, SD = 10.4) than students (*M* = 2.1 years, SD = 2.1), while 7.6% had no work experience.

#### 2.3.2. Sample for test-retest reliability

In order to estimate test-retest reliability, the MVSQ was re-issued to 166 students 12 weeks after they had received written feedback reports of their results as part of a complementary psychology course. *N* = 72 (49 women, 68.1%) completed the questionnaire again. Mean time interval between test and retest was 15 weeks and ranged from 12 to 23 weeks (SD = 2.6). Their mean age at first test administration was 23.7 years (SD = 2.6; range = 19–33) and almost all of the participants were German (71, 98.6%). 29 (40.3%) participants were business students, 8 (11.1%) majored in computer science and 35 (48.6%) had other major subjects. Their overall mean work experience was 1.7 years (SD = 2).

#### 2.3.3. Sample for convergent validity

To assess construct validity, we administered the SVS and the MVSQ to a sample of 102 students (70 women, 68.6%) of which 50 were also part of the test-retest sample. The mean age was 23.3 years (SD = 2.7; range = 18–33), 98 persons were German (96.1%) and, regarding major subjects, 40 (39.2%) were business students, 12 (11.8%) majored in computer science and 50 (49%) majored in other subjects.

#### 2.3.4. Sample for concurrent validity

With the aim of achieving sufficient power when testing for differences in value systems between employees with similar job characteristics, we computed mean trait scores for departmental affiliations only if group size was *n* > 100. The composite sample contained three categories of departments which fulfilled this requirement: Sales (*n* = 185), research and development (R&D, *n* = 161) as well as personnel (*n* = 134). The factorial design of ANOVAs is nonorthogonal with unequal sample sizes, meaning main effects and interactions are not independent and probability of a Type I error is increased. Therefore, we chose to randomly delete cases in sales and R&D samples until they matched the personnel sample size (Tabachnick and Fidell, 2007). Consequently, the whole sample comprised *N* = 402 employees. Their mean age was 36.6 years (SD = 10.4; range = 20–63) and 163 were women (40.5%). The overall mean job experience amounted to 12.4 years (SD = 10.1).

### 2.4. Data analysis

#### 2.4.1. Thurstonian IRT modeling

A Thurstonian Item-Response Theory (TIRT) model (Brown and Maydeu-Olivares, 2011, 2013) was estimated using the kcirt package (Zes et al., 2014) in the R environment Version 3.3.0 (R Core Team, 2016). In contrast to the procedure described by Brown and Maydeu-Olivares (2012), in which model parameters and scores are estimated consecutively, in kcirt they are estimated jointly by applying a least squares expectation-expectation algorithm and metaheuristic stochastic search (MSS). Both estimation algorithms make use of shrinkage, which is a method that constrains coefficient estimates (shrinks them toward zero) in order to improve model fit by reducing their variance (James et al., 2014). Tuning parameters were determined in a simulation study and were selected as sufficiently small (0.001–0.1) to achieve robust and unbiased estimates, but large enough to result in convergence of the estimation algorithm. Because of using shrinkage, neither uniquenesses nor loadings needed to be fixed.

Further, we conducted a cross-method comparison of IRT scores and classical test theory (CTT) scored forced-choice responses (CTT scores) to check for plausibility for two reasons: (1) Estimating a TIRT model that contains seven items per block using DWLS and MAP estimator (Brown and Maydeu-Olivares, 2012) would lead to a gross overestimation of the item information functions and thus distorted IRT scores (which is one of the reasons why we used kcirt). (2) To our knowledge, there are no publications applying kcirt to models with seven items per block. We thereby expected profile scores to differ between CTT and IRT scores both in terms of average profile score and of rank correlation (Brown and Maydeu-Olivares, 2013).

#### 2.4.2. Model fit

As large TIRT models are too complex to determine model fit on a mathematical basis (Brown and Maydeu-Olivares, 2013), we determined model fit to the data in a simulation study, estimating Root Mean Square Error (RMSE) as a measure of average deviation of an estimated model to a simulated (*true*) model. The *true* model in this case was the model that was fitted to the original data and the estimated model was a model that was fitted to randomly-generated rank data based on original model parameters (Zes et al., 2014). The RMSE was calculated as the mean deviation of standardized loadings, utilities and score intercorrelations. In order to preclude distortions due to random sample generation within the simulation, we replicated the RMSE computation and computed a mean RMSE over all replications. The number of replications was set to 10 which in Monte-Carlo Studies in IRT can be considered large enough regarding the sample size of *n* > 1000 (Harwell et al., 1996). To facilitate interpretation of the RMSE in this case, the coefficient was standardized to the same metric as the model parameters.

#### 2.4.3. Measurement precision

In IRT, the standard error of measurement is not assumed to be uniform across the latent trait continuum (Embretson and Reise, 2000). Consequently, precision of measurement varies across different trait levels and it is not meaningful to calculate an item inter-relatedness coefficient such as Cronbach's α. Alternatively, a sample-based approach of reliability estimation can be used that estimates test precision via simulation, referred to as marginal reliability (Green et al., 1984; Ayala, 2013; Brown and Croudace, 2015) or empirical reliability (Maydeu-Olivares and Brown, 2010; Brown and Maydeu-Olivares, 2011). Here, the procedure consisted of five steps: (1) We fitted a TIRT model to the original data. (2) We generated *n* = 1,217 sets of scores correlated as in the original model as well as respective random error terms (uniquenesses). (3) We then calculated the corresponding response data set using original loadings and utilities. These simulated scores can be referred to as *true* scores as the perfectly matching response data set is known. (4) A TIRT model is fitted to this simulated data set. (5) Reliability was then computed as the squared correlation of true scores and estimated scores. All five steps can be done with the help of various functions of the kcirt package. We also replicated this procedure 10 times in order to preclude distortions due to effects of random realization of simulated scores and random errors, and we calculated both median and range of each trait's empirical reliability estimate.

#### 2.4.4. Penalized maximum likelihood estimation

As a note to penalized maximum likelihood methods (such as MSS), it is important to notice that parameter estimation is sensitive to the penalty size (Hastie et al., 2013). Thus, both RMSE and empirical reliability estimates computed with kcirt were affected by the selection of tuning parameters which are the penalty parameters within the MSS algorithm. In a simulation study we found that if tuning parameters were set too small, model parameters were not differentiated enough (shrunk too little), RMSE was poor and empirical reliability was overestimated. In addition, with extremely small penalties the estimation did not converge. In contrast, shrinkage terms that were set too large yielded underestimted reliabilities (and also poor fit). Our final selection of tuning parameters showed an average of less than 2% of distortion of empirical reliability estimates over ten replications and a mean RMSE of 0.07 (ranging from 0.06 to 0.11). Given the absolute magnitudes of utilities (*M* = 0.46), factor loadings (*M* = 1.3), and score intercorrelations (*M* = 0.26), and taking into account that RMSE is on the same metric as the dependant variables, this can be considered an acceptable fit.

IRT scores that were used to compute test-retest reliability, convergent and concurrent validity were estimated using the combined data set of all samples (*N* = 1,815 participants). The rationale behind this decision was that, with larger sample sizes, estimation of TIRT model parameters and IRT scores becomes more robust. We noted that sample sizes of *N* < 500 were too small to yield robust estimation of a MVSQ TIRT model and respective IRT scores with the kcirt package.

#### 2.4.5. Validity analysis

We examined construct validity through a variety of analyses. Scale score intercorrelations offered insight into the relations between value systems. Correlations of value systems with age were expected to show small coefficients as found in other studies about values and age (Feather, 1977; Cherrington et al., 1979). Convergent and discriminant validity was analyzed using a multitrait-multimethod (MTMM) matrix (Campbell and Fiske, 1959) comparing latent traits measured by the MVSQ and the Schwartz Value Survey (SVS), one of the most widespread measure of value systems (Schwartz, 2012). Moreover, we assessed gender differences based on hypotheses derived from previous research by Schwartz and criterion-related validity comparing the average scores of employees in different departments.

Within the MTMM analysis, monotrait-heteromethod (MTHM) coefficients indicate convergent validity. These should be significantly different from zero and substantial in magnitude (Byrne and Goffin, 1993), and according to Fiske and Campbell (1992), typically range between 0.30 and 0.50. Further, convergent validities should be higher than both heterotrait-heteromethod (HTHM) and heterotrait-monomethod (HTMM) correlations to establish discriminant validity (Campbell and Fiske, 1959). In accordance with Bagozzi and Yi (1991) and Byrne and Goffin (1993), discriminant validity is considered high when less than 5% of discriminant validity coefficients exceed convergent validity coefficients, moderate when the percentage lies between 6% and 33%, and low when violations surpass 33%. We investigated relationships between MVSQ and SVS scale scores for evidence on the validity of the categorization of MVSQ value systems into seven factors. Based on theoretical similarities, we expected that MVSQ scales would correlate positively with specific SVS scales as shown in Table 3,^1^ (Strack, 2011). SVS reliability was estimated by Cronbach's α.

**Table 3:** Conceptual similarity of value systems measured by MVSQ and SVS.

**MVSQ**	**SVS**
Preserving	Tradition
Doing	Power
Complying	Conformity, Security
Achieving	Achievement
Harmonizing	Benevolence
Understanding	Self-direction
Sustaining	Universalism

*Schwartz' value systems Hedonism and Stimulation were expected to be unrelated to Graves' value system conceptualizations*.

Based on the findings in a large sample from Schwartz and Rubel (Schwartz and Rubel, 2005), we expected some value system preferences to differ regarding gender. Value systems **Doing**, **Achieving** and **Understanding** were hypothesized to show higher scores for men and **Harmonizing** and **Sustaining** for women.

Further, we examined criterion-related validity as concurrent validity and hypothesized that mean value systems scores of employees differ systematically between departments. We therefore compared mean value system scale scores of members of R&D, sales and personnel departments. Different departments, on average, have different job characteristics and thus attract people with congruent value system preferences (Rousseau, 1978). A main characteristic of jobs in R&D departments is innovativeness (e.g., Judge et al., 1997; Elkins and Keller, 2003). Conceptually the value system **Understanding** matches this feature of R&D departments as people with high standings on this value system enjoy engaging in deep theorizing and innovative thinking. Thus, we hypothesized that members of R&D departments on average score higher on **Understanding**. In contrast, sales jobs require high degrees of flexibility and goal-orientation (Dubinsky et al., 1986) these characteristics are congruent with high preferences for the value system **Achieving**. Obviously, motivation and success of a salesperson depends on many factors, such as the type of product sold, sales skills, and experience. Nevertheless, we expected that, on average, i.e., across branches and companies, members of sales departments score higher on this value system. Further, a major function of personnel departments is mediating between different stakeholders and the personnel department is linked to all departments of an organization because all managers and employees depend upon activities of the personnel department (Tsui, 1984). We therefore expected the value system **Harmonizing** to be strongly related to employees of personnel departments, because people with high standings on **Harmonizing** seek harmony with others and enjoy engaging with others in general.

## 3. Results

On average, it took respondents 29.35 min (median = 25.97 min) to complete the MVSQ's 20 blocks. Thus, respondents needed on average 12.58 s to rank one item and 1.47 min to establish the rank order of all seven items within one block. In comparison, mean processing time of SVS' 57 rating tasks was 12.32 min (median = 10.18 min) and the average time to rate one item was 12.97 s.

### 3.1. TIRT model parameters

Table 4 shows factor loadings that resulted from the TIRT model estimation. This parameter reveals an item's discriminatory power in comparison to other items. As standard errors were not available from the estimation procedure implemented in kcirt, evaluation of estimates was limited to theoretical considerations and comparison with other loadings. Most loadings showed values considerably different to zero, suggesting that these items are indicators of the latent traits as intended by design. Across traits, there was one trait with notably weaker loadings (**Achieving**) which showed two loadings above zero, while being negatively keyed. In addition, block 6 and block 18 showed a number of poor loadings.

**Table 4:** MVSQ item factor loadings (see Table 2 for mapping of blocks to situations; blocks 1 – 10 contain positively keyed items, blocks 11 – 20 negatively keyed items).

**Value system**	**Situation and Block**											***M***
	**Task and goal striving**			**Performance**	**Work env**.	**Recognition**		**Conflict**		**Work load**	**Decision making**	
	**1**	**7**	**9**	**2**	**3**	**4**		**5**	**6**	**8**	**10**	
Preserving	1.25	0.41	1.51	0.85	2.23	0.73		1.05	1.54	1.69	0.62	1.19
Doing	1.74	1.09	1.57	0.86	2.03	1.00		1.79	0.83	1.35	0.92	1.32
Complying	1.90	1.76	1.72	1.88	1.51	1.54		1.61	0.90	1.57	0.93	1.53
Achieving	1.36	1.45	1.29	1.68	1.32	1.80		0.50	0.44	1.46	1.05	1.24
Harmonizing	0.88	1.06	1.36	1.71	1.09	0.74		1.12	0.81	1.11	1.71	1.16
Understanding	2.36	1.10	1.97	1.63	0.54	1.41		1.04	0.76	1.17	0.53	1.25
Sustaining	0.99	2.35	2.79	1.16	1.56	1.90		1.48	0.27	1.12	2.33	1.60
	**11**	**19**	**20**	**15**	**13**	**12**	**14**	**17**		**16**	**18**	***M***
Preserving	−1.61	−1.70	−1.69	−1.51	−1.21	−1.22	−1.01	−1.11		−1.86	−0.88	−1.38
Doing	−1.98	−2.24	−0.84	−1.61	−1.09	−1.19	−1.00	−1.57		−1.96	−0.45	−1.39
Complying	−1.49	−1.87	−2.06	−2.34	−1.14	−1.37	−0.95	−1.21		−2.03	−1.01	−1.55
Achieving	−2.35	−2.29	0.25	−2.08	−1.28	−0.10	−1.53	0.08		−0.48	−1.23	−1.10
Harmonizing	−2.22	−1.59	−1.45	−1.63	−1.54	−1.88	−0.88	−1.01		−1.63	−0.51	−1.43
Understanding	−2.35	−2.38	−1.29	−1.00	−1.09	−0.97	−0.92	−1.08		−0.75	−0.61	−1.24
Sustaining	−1.55	−1.16	−0.43	−0.73	−1.25	−1.32	−1.12	−0.93		−0.39	−0.59	−0.95

*N = 1,217*.

Table 5 shows utilities as estimated by the TIRT model. Utilities indicate item difficulty, with a high utility indicating a high probability of the item being ranked above other items. Concerning the first 10 blocks (positively keyed items), the value systems **Harmonizing** and **Understanding** showed comparably high utilities, while relatively low utilities were found for **Preserving** and **Sustaining**. These results suggest a moderate imbalance regarding the areas of the latent trait continuums at which these value systems are being measured. With regard to blocks 11–20 (negatively keyed items), item utilities were found to be more homogeneous overall.

**Table 5:** MVSQ item utilities (see Table 2 for mapping of blocks to situations; blocks 1 – 10 contain positively keyed items, blocks 11 – 20 negatively keyed items).

**Value system**	**Situation and Block**											***M***
	**Task and goal striving**			**Performance**	**Work env**.	**Recognition**		**Conflict**		**Work load**	**Decision making**	
	**1**	**7**	**9**	**2**	**3**	**4**		**5**	**6**	**8**	**10**	
Preserving	−1.09	0.26	−0.60	−0.66	−0.15	−1.54		−0.51	−1.49	−0.48	−0.41	−0.67
Doing	−0.04	−0.46	−0.12	−0.52	0.42	0.25		0.38	−1.10	0.08	−0.51	−0.16
Complying	−0.11	0.29	−1.31	−0.28	0.42	−0.65		−0.69	0.22	0.42	−0.22	−0.19
Achieving	0.06	0.38	0.43	−0.55	−1.52	0.60		0.57	0.26	0.35	−0.23	0.03
Harmonizing	0.97	−0.16	0.89	0.76	0.70	0.62		0.81	0.68	−0.27	0.37	0.54
Understanding	0.83	0.77	0.85	0.97	0.76	0.99		0.50	1.19	0.62	1.52	0.90
Sustaining	−0.63	−0.84	−0.24	0.17	−0.61	−0.34		−0.79	0.24	−0.70	−0.27	−0.40
	**11**	**19**	**20**	**15**	**13**	**12**	**14**	**17**		**16**	**18**	***M***
Preserving	0.80	0.67	0.22	−0.13	0.14	0.23	0.27	0.04		0.68	0.12	0.30
Doing	−0.40	−0.15	−0.20	0.31	0.64	0.33	0.30	0.09		−1.00	0.53	0.04
Complying	0.59	0.58	0.08	0.44	0.53	−0.10	−0.08	−0.33		0.20	0.19	0.21
Achieving	0.19	0.13	0.31	0.23	−0.14	0.52	−0.55	0.14		−0.60	−0.10	0.01
Harmonizing	0.11	−0.49	0.32	0.34	−0.25	−0.50	0.01	0.05		0.29	−0.23	−0.03
Understanding	−0.90	−0.71	−0.29	−0.81	−0.19	−0.32	0.31	−0.05		0.25	0.22	−0.25
Sustaining	−0.18	0.30	−0.44	−0.36	−0.59	−0.37	−0.25	0.22		0.30	−0.74	−0.21

*N = 1,217*.

### 3.2. Reliability

Table 6 summarizes reliability estimates (empirical reliability ρ and test-retest reliability *r*_*tt*_) of scale scores. Median empirical reliabilities ranged between 0.74 and 0.84 (*M* = 0.78). The estimates ranged between 0.02 and 0.04. This spread can be considered as narrow, indicating a good fit of tuning parameters and low variance in estimation. Test-retest reliabilities were slightly higher and ranged between 0.77 and 0.90 with a mean of 0.83.

**Table 6:** MVSQ empirical (ρ) and test-retest reliabilities (*r*_*tt*_).

**Value system**	**ρ**		***r*_*tt*_**
	**Median**	**Range**	
Preserving	0.75	0.73–0.77	0.79
Doing	0.80	0.79–0.82	0.78
Complying	0.84	0.83–0.85	0.88
Achieving	0.77	0.75–0.79	0.81
Harmonizing	0.74	0.72–0.77	0.77
Understanding	0.75	0.73–0.76	0.90
Sustaining	0.79	0.77–0.81	0.88

*Median and range of empirical reliabilities (ρ) were calculated over ten replications; N_ρ_ = 1,217; N_tt_ = 72*.

### 3.3. Cross-method comparison between CTT and IRT scores

**Figure 1:**
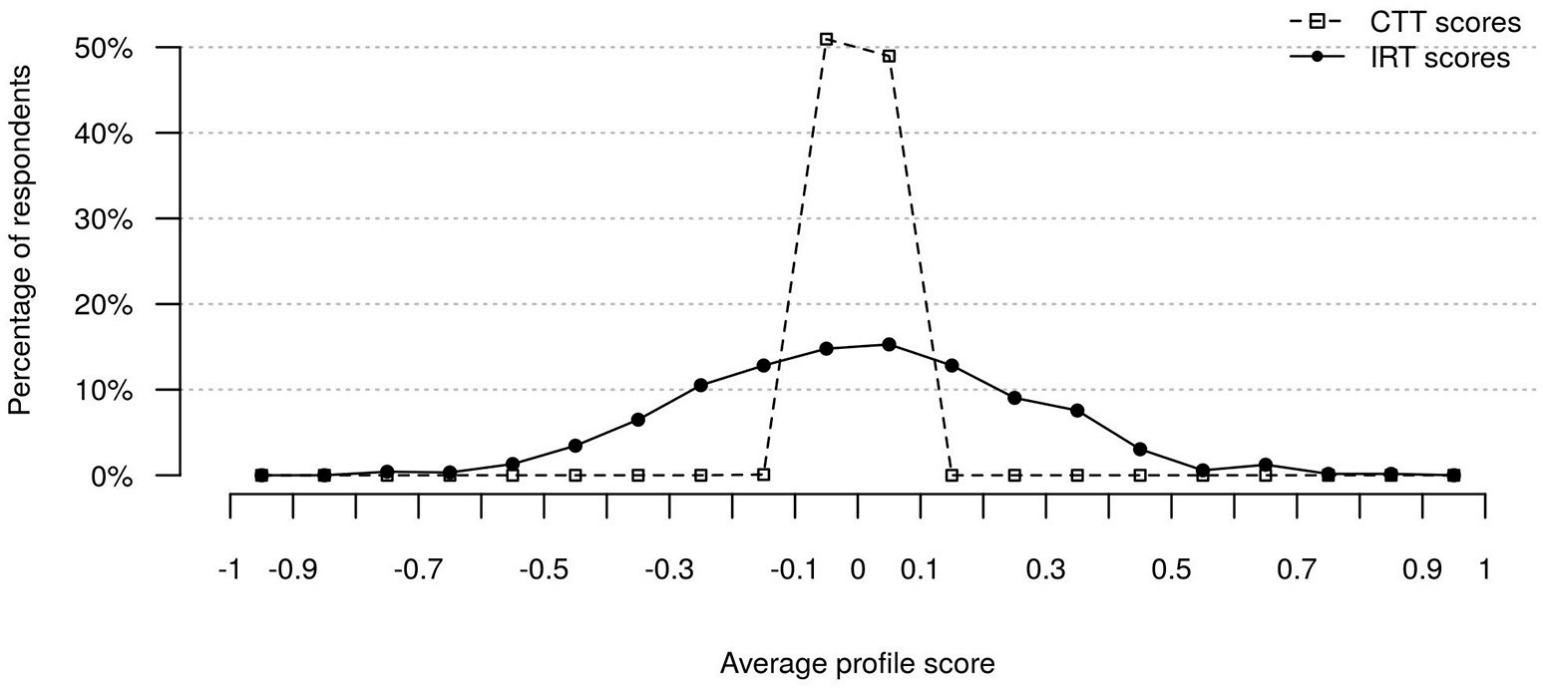
Distributions of average MVSQ profile scores: CTT versus IRT scores; *N* = 1,217.

The average intercorrelation of ipsative scale scores is determined by block size, with expected average *r* = −0.17 for blocks with seven items. In comparison, the intercorrelations of IRT-derived value system scores shown in Table 7 were substantially smaller, with an average intercorrelation of *r* = −0.09. Further, Figure 1 illustrates average profile scores across all value systems. Both CTT and IRT scores were transformed to *z*-scores to facilitate meaningful comparison. It can be seen that CTT scoring produced almost equal average profile scores for all respondents due to ipsativity (average CTT z-scores ranged from −0.11 to 0.1, SD = 0.03). In contrast, average IRT scores showed a wide spread, with scores ranging from −0.80 to 0.85 (SD = 0.26). Finally, Spearmans rank correlation coefficients were computed between IRT and CTT scores of each participant to check for rank-order similarity. Rank correlations ranged from 0.29 to 1 (median = 0.96; *M* = 0.94; SD = 0.08). Thus, although the average correlations were high, some profiles showed substantial differences between type of scores.

**Table 7:** MVSQ score inter-correlations.

	**PR**	**DO**	**CO**	**AC**	**HA**	**UN**
Preserving (PR)						
Doing (DO)	−0.31					
Complying (CO)	0.55	−0.24				
Achieving (AC)	−0.26	0.36	−0.07			
Harmonizing (HA)	0.32	−0.43	0.11	−0.46		
Understanding (UN)	−0.41	−0.05	−0.29	−0.02	−0.12	
Sustaining (SU)	−0.15	−0.19	−0.28	−0.35	0.20	0.19

*N = 1,217*.

### 3.4. Validity

#### 3.4.1. Value system intercorrelations

Value system scale score intercorrelations summarized in Table 7 showed a wide variation of positive and negative scale score associations (between −0.46 and 0.55). Overall, a pattern can be recognized between two groups of value systems. **Preserving**, **Complying** and **Harmonizing** form a positively correlated group of value systems (mean *r* after Fisher's *z* transformation = 0.34), while they were found to be negatively correlated (mean *r* = −0.29) with a second group of value system scales comprising **Doing**, **Achieving** and **Understanding**. The mean of correlations within the latter group of value systems was *r* = 0.10 with two correlations close to zero and one considerably higher. Moreover, **Sustaining** value system scores were positively correlated with **Harmonizing** and **Understanding** (mean *r* = 0.19), and negatively correlated with **Preserving**, **Doing**, **Complying** and **Achieving** (mean *r* = −0.24).

#### 3.4.2. Value systems, gender and age

We examined gender differences regarding value system scores by conducting a series of exploratory independent samples *t*-tests. These revealed that women scored significantly higher than men on value systems **Preserving**, *t*_(1, 062)_ = 7.43, *p* < 0.001, **Complying**, *t*_(1, 126)_ = 5.58, *p* < 0.001 and **Harmonizing**, *t*_(1, 123)_ = 7.46, *p* < 0.001. Men obtained significantly higher scores on value systems **Doing**, *t*_(1, 138)_ = 6.81, *p* < 0.001, **Achieving**, *t*_(1, 117)_ = 5.83, *p* < 0.001 and **Understanding**, *t*_(1, 098)_ = 5.26, *p* < 0.001. No difference was found for **Sustaining**.

Correlations of trait scores with age were small (all *r* < |0.30|); only three associations were of noticeable strength: **Preserving**, **Complying** tended to decrease with age (*r* = −0.24 and *r* = −0.21, respectively), whereas higher levels of **Doing** were observed with increasing age (*r* = 0.29).

#### 3.4.3. Convergent and discriminant validity

We produced a MTMM matrix as shown in Table 8. Following the previously specified criteria, strong evidence of convergent validity was found for **Doing** (0.40), **Complying** (0.32 and 0.25), **Achieving** (0.54), **Harmonizing** (0.34), **Understanding** (0.41) and **Sustaining** (0.57). **Preserving** (0.15) showed no significantly positive MTHM correlations. As for discriminant validity, 5 (4.8%) out of all 104 HTHM coefficients (range from –0.49 to 0.53; *M* = −0.08), omitting those involving SVS value systems Hedonism and Stimulation, exceeded respective validity coefficients. Regarding HTMM correlations (range from −0.52 to 0.67; *M* = 0.13), 29 (27.9%) did not meet the criterion of being lower than respective validity coefficients. Thus, discriminant validity was good regarding HTHM coefficients, while it was moderate regarding HTMM coefficients. As for value systems Hedonism and Stimulation that were hypothesized to have no systematic relations to MVSQ value systems, correlations of MVSQ sclaes with Hedonism were not significant, whereas Stimulation showed a significant positive correlation with the MVSQ scale **Doing** (0.25) and two significant negative correlations with MVSQ scales **Preserving** (−0.33) and **Complying** (−0.34).

**Table 8:** Multitrait-multimethod matrix for value systems using MVSQ and SVS.

**Value system**	**MVSQ**							**SVS**									
	**PR**	**DO**	**CO**	**AC**	**HA**	**UN**	**SU**	**TR**	**PO**	**CO**	**SE**	**AC**	**BE**	**SD**	**UN**	**HE**	**ST**
**MVSQ**																	
PR	( )																
DO	−0.31	( )															
CO	0.67	−0.25	( )														
AC	−0.15	0.35	0.04	( )													
HA	0.37	−0.44	0.16	−0.52	( )												
UN	−0.43	−0.05	−0.29	−0.19	−0.22	( )											
SU	−0.11	−0.10	−0.31	−0.37	0.24	0.05	( )										
**SVS**																	
TR	**0.15**	−0.09	0.28	−0.09	0.07	0.01	−0.14	(0.60)									
PO	−0.26	**0.40**	−0.15	0.53	−0.42	−0.08	−0.31	0.14	(0.73)								
CO	0.12	0.00	**0.32**	0.28	−0.13	−0.20	−0.29	0.59	0.41	(0.64)							
SE	0.19	0.00	**0.25**	0.05	0.04	−0.33	−0.11	0.54	0.29	0.65	(0.58)						
AC	−0.36	0.29	−0.12	**0.54**	−0.44	0.04	−0.33	0.16	0.65	0.45	0.21	(0.72)					
BE	0.06	−0.22	0.04	−0.32	**0.34**	−0.07	0.21	0.54	−0.10	0.36	0.37	−0.11	(0.62)				
SD	−0.49	0.14	−0.34	−0.17	−0.17	**0.41**	0.16	0.22	0.08	0.03	0.10	0.25	0.32	(0.55)			
UN	−0.05	−0.07	−0.19	−0.47	0.29	−0.02	**0.57**	0.31	−0.18	0.01	0.29	−0.11	0.55	0.44	(0.80)		
HE	−0.02	0.03	−0.16	0.03	−0.07	−0.19	−0.11	−0.03	0.20	0.04	0.26	0.17	0.00	0.13	0.16	(0.70)	
ST	−0.33	0.25	−0.34	0.08	−0.15	0.03	0.17	−0.11	0.10	−0.05	−0.05	0.28	−0.04	0.47	0.32	0.34	(0.75)

*Following Campbell and Fiske (1959), validity coefficients (monotrait-heteromethod correlations) are marked in bold typeface; MVSQ, Motivational Value Systems Questionnaire; SVS, Schwartz Value Survey; for the MVSQ, PR, Preserving, DO, Doing, CO, Complying, AC, Achieving, HA, Harmonizing, UN, Understanding, SU, Sustaining; for the SVS, TR, Tradition, PO, Power, CO, Conformity, SE, Security, AC, Achievement, BE, Benevolence, SD, Self-direction, UN, Universalism, HE, Hedonism, ST, Stimulation. Coefficients in parenthesis reflect Cronbachs α (SVS); Empirical reliability coefficients (MVSQ) could not be estimated due to insufficient sample size thus empty parenthesis; Values > 0.19 are statistically significant (p < 0.05), values > 0.26 (p < 0.01), and values > 0.33 (p < 0.001); N = 102*.

#### 3.4.4. Value systems preferences and departmental affiliation

**Figure 2:**
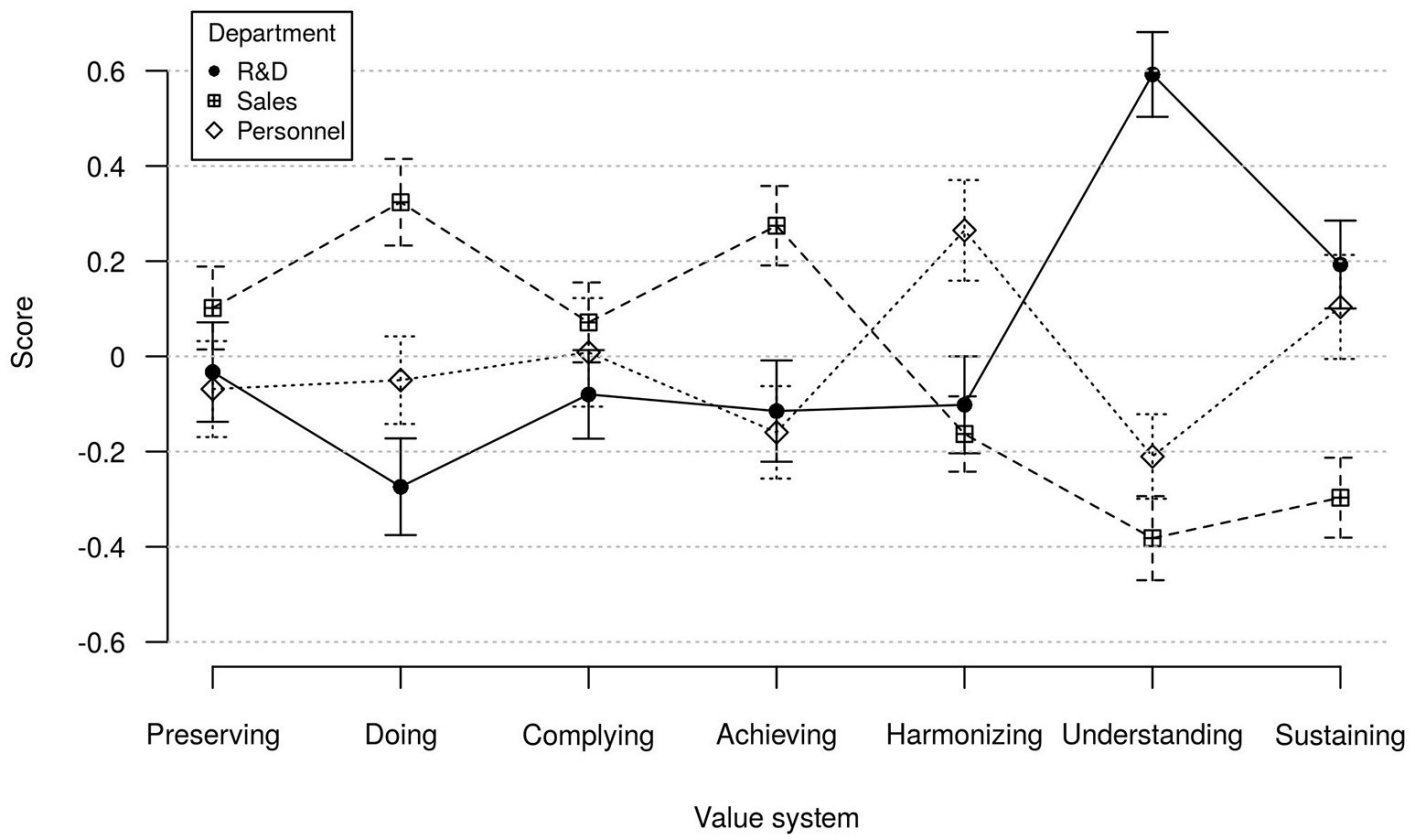
Mean MVSQ value system scores of members of R&D, sales and personnel departments. Error bars show 95% confidence intervals; *N* = 402.

Figure 2 shows average scores for value systems of employees in R&D, sales and personnel departments. We computed separate ANOVAs to test for differences in scale scores of value systems **Achieving**, **Harmonizing** and **Understanding** between departmental affiliations. All three ANOVAs showed significant main effects: **Achieving**, *F*_(2, 399)_ = 7.9, *p* < 0.001, η^2^ = 0.04; **Harmonizing**, *F*_(2, 261)_ = 7.08, *p* < 0.01, η^2^ = 0.04; **Understanding**, *F*_(2, 399)_ = 44.02, *p* < 0.001, η^2^ = 0.18. The *F*-value for **Harmonizing** was computed using robust *F*-test (Welch, 1951) due to inhomogeneity of variances. Further we computed Tukey HSD-tests which revealed the following mean differences as hypothesized: Sales people scored significantly higher on **Achieving** compared to both R&D (*p* < 0.01) and personnel employees (*p* < 0.001). Members of personnel departments surpassed sales and R&D employees (both *p* < 0.01) on **Harmonizing**, and R&D employees scored higher on **Understanding** than members of sales and personnel departments (both *p* < .001).

Additionally, we found differences for value systems **Doing**, *F*_(2, 399)_ = 12.95, *p* < 0.001, η^2^ = 0.06, and **Sustaining**, *F*_(2, 263)_ = 11.13, *p* < 0.001, η^2^ = 0.05. Here, the *F*-value for **Sustaining** was computed using a robust *F*-test due to inhomogeneity of variances. Sales people had significantly higher standings on **Doing** than R&D (*p* < 0.001) and personnel employees (*p* < 0.01), but scored significantly lower than both R&D (*p* < 0.001) and personnel employees (*p* < 0.01) on **Sustaining**.

## 4. Discussion

The present study introduced the MVSQ, a questionnaire for the assessment of value system preferences utilizing a forced-choice response format. Results of fitting a Thurstonian IRT model suitable for modeling ipsative data provided support for the scale's factorial structure and for acceptable item characteristics. Empirical reliability estimates resulted in coefficients between 0.74 and 0.84 for the individual value system scale scores, suggesting acceptable-to-good measurement precision. Intercorrelations between scale scores, gender differences and correlations with age showed plausible associations. Finally, correlations with a similar measure of value systems, as well as differences between mean value preferences of employees working in R&D, sales and personnel departments, support validity of the MVSQ scales.

### 4.1. Design of the questionnaire and IRT modeling

The MVSQ consists of 20 blocks, each containing seven unidirectionally keyed items. Referring to Brown and Maydeu-Olivares (2011, 2013), this structure is not optimal to recover true trait standings, as (a) items are keyed in the same direction and (b) some intertrait correlations are substantially positive. Despite these adverse characteristics, latent trait and item parameter recovery was sufficient to yield both meaningful model parameters and satisfying measurement precision.

The questionnaire's design presented considerable challenges for psychometric modeling and model estimation, as the determination of adequate tuning parameters required the estimation of hundreds of models (one for each set of tuning parameters), each with a computing time of between approximately 12 and 14 h (using 4 cpus). Besides reasonable model fit indices, IRT modeling can be considered successful, because MVSQ scoring by the TIRT model effectively dealt with restrictions that result from ipsativity of data. Scale score intercorrelations were significantly lower compared to classically scored forced-choice responses and were thus no longer determined by the number of traits measured. In addition, rank correlations showed notable differences in score profiles between IRT and CTT scores regarding ordering of traits. Both the differences between IRT and CTT ordering of traits and the mean profile scores reported here resemble those reported in Brown and Maydeu-Olivares (2013), which further supports the validity of the fitted TIRT model.

The cognitive load imposed on respondents is a limiting factor of block size when ranking items (Brown and Maydeu-Olivares, 2011). However, results of the TIRT model applied here, particularly MVSQ-items' loadings on the latent factors and empirical reliabilities, suggest that sorting seven items per block presents no unreasonable cognitive load. Furthermore, mean processing time of SVS per rating und MVSQ per ranking tasks, which were practically equal, can be interpreted in such way that ranking seven items per block does not overcharge respondents.

Overall, our findings demonstrate that a block size of seven is feasible, both for respondents completing the questionnaire and the researcher modeling an IRT model. Larger blocks come with the advantage of generating exponentially more binary comparisons, and thus information about trait standings, with each item added (Brown and Maydeu-Olivares, 2011). Therefore, larger blocks can compensate for a smaller number of blocks. A future investigation should be directed into the effects of reducing blocks on model fit and measurement precision.

### 4.2. Precision of measurement

The MVSQ should be difficult to be intentionally distorted, as FC measures are, in general, more robust against response sets than single stimulus measures (Jackson et al., 2000; Cheung and Chan, 2002; Martin et al., 2002; Christiansen et al., 2005; Vasilopoulos et al., 2006; Bartram, 2007). Nevertheless, future studies should investigate effects of faking responses on scale scores. While the MVSQ's specification is beneficial for reduced susceptibility to intentional distortion, it is not optimal for maximizing measurement precision. Brown and Maydeu-Olivares (2011, 2013) showed that FC questionnaires measuring positively-correlated traits with unidirectionally keyed items have a reduced ability to recover absolute trait standings of individuals which, in turn, leads to weaker reliabilities. However, empirical reliability estimates of all traits were acceptable or good. Especially considering the broadness of the construct with each value system being operationalized as states and behaviors of a variety of work situations, reliability estimates seem strong. Further, test-retest reliabilities suggest at least medium-term temporal stability, although small sample size (*N* = 72) can be criticized. Future studies need to be done, not only with larger Ns but also to test for different retest intervals and different cohorts.

### 4.3. Validation studies

Scale score intercorrelations are overall plausible, without any of the correlations being extreme (maximum magnitude *r* = 0.55), thus indicating that the latent variables are empirically separable. The pattern of scale score intercorrelations corresponds to Graves' theorizing. He categorized value systems in one of two groups: (a) express self or (b) sacrifice self (Graves, 1971c). **Doing**, **Achieving**, and **Understanding** pertain to the express self group and **Preserving**, **Complying**, **Harmonizing**, and **Sustaining** belong to the sacrifice self group. The scale score intercorrelations found in our study are largely in line with this theoretical notion. Thus, the pattern of intercorrelations found in our study is largely consistent with Graves' categorization into express self and sacrifice self value systems (Graves, 1971c) and, therefore, supports construct validity of the MVSQ. Further, this categorization of value systems showed similarities to gender differences as men scored higher on all express self value systems and women scored higher on three of the four sacrifice self value systems. Apart from that, gender differences were as hypothesized for men. For women, the expected result for value system **Sustaining** was not consistent with findings by Schwartz and Rubel (2005). Considering the lack of studies on the relation between gender and value systems based on Graves' theory, results can only be seen as exploratory, and more studies are required. Associations of value systems with age were as hypothesized, i.e., correlations were small and in line with previous findings.

Compared to other results of MTMM matrices involving the SVS (Schwartz et al., 2001; Schwartz, 2005), validity coefficients of the current analysis were slightly lower which was to be expected given that the two measures differ clearly in terms of conceptualization of value systems. For instance, within the Schwartz (1994) framework, the value *Status* belongs to value system Power, but in Graves (1974) theory it is associated with **Achieving**. Nevertheless, six value systems showed validity coefficients as hypothesized and additionally had good to moderate discriminant validity. The validity of the value system **Preserving** remains unclear as it did not show a significant validity coefficient and a high intertrait correlation with **Complying** (0.67), suggesting **Preserving** items need further adjustment. On the other hand, the SVS value system Tradition (parallel to **Preserving**) showed a noticeable overlap with other SVS value systems, i.e. with Conformity (0.59), Security and Benevolence (both 0.54) which indicates some fuzziness of constructs. As for the completeness of the nomological network, the MVSQ does not represent the value system Hedonism which shows no significant correlations to any MVSQ value system. In contrast to value systems, hedonism is not seen as a primary source of motivation in the context of work motivation (Steers et al., 2004; Pinder, 2008). Instead it is understood as one of several principles of motivation (Weiner, 1992; Koole and Kuhl, 2008; Deckers, 2015). Future research should deal with the question of whether hedonism qualifies as a value system. To sum up, our results largely support validity of MVSQ scores. Nevertheless future studies should include additional values questionnaires such as the Portrait Values Questionnaire (PVQ Schwartz et al., 2001), the Rokeach Value Survey (RVS Rokeach, 1973) or the Hofstede's work-related cultural dimensions (Hofstede, 1980). In addition, the relationship between value systems and basic principles of motivation such as hedonism, homeostasis, or approach and avoidance motivation should be investigated in order to further examine the meaningfulness of the value system categorization by Graves.

With regard to differences in average value system preferences between departments, we found hypothesized differences in average scores for R&D, sales and personnel departments. This finding is consistent with the assumption that value systems preferences are associated with specific departmental characteristics, and that the MVSQ can be used to detect such differences. Nevertheless, because of restrictions in sample size our findings are limited to these three departments only. A research gap has emerged when searching for value system-related department characteristics with only few articles dealing with typical characteristics of work content in departments. Assuming that further associations between value systems and departments exist, future studies might yield results that could be applied to the benefit of both organizations and employees. Establishing a theoretical framework of congruence between value systems and departmental characteristics on the basis of empirical results would make for a useful model to systematically advise jobseekers regarding well-fitting jobs.

### 4.4. Limitations and future directions

Several limitations need to be addressed. First, this study is based on a convenience sample which, on the upside contained data from professionals not related to the universitarian environment. On the downside these were acquired through consultation projects which requires openness to test-taking on behalf of the customers and, therefore, might have introduced self-selection bias. When developing test norms in the future, probability sampling and larger samples are needed. Second, the kcirt package does not provide a standardized measure of model fit. The development of such a measure would facilitate comparability across studies and make the estimation algorithm more readily applicable. Third, despite the encouraging results of our validation studies, more needs to be done to support the validity of MVSQ scores beyond initial evidence. The MTMM analysis had several weaknesses: (a) lack of a true parallel instrument, (b) unavailability of MVSQ reliability estimates, and (c) low reliability of SVS (alphas ranged from 0.55 to 0.80; *M* = 0.67). Ideally, an MTMM analysis would be conducted with a truly parallel measure of value systems based on Graves' theory, and with sample sizes sufficiently large to estimate measurement precision. Considering findings on value system dominance in sales, R&D and personnel departments, more evidence on dominating value systems in different departments should be collected to allow wider generalizability across departments. Further, it would be interesting to assess implications of congruence between value systems and job characteristics on person-centered variables such as mood, satisfaction and commitment. Fourth, MVSQ items should be revised to improve on low factor loadings and item utilities. This is particularly relevant for MVSQ blocks 6, 10, and 18, and items with factor loadings close to zero. However, improving psychometric item characteristics by revising item wording is notoriously difficult with forced-choice questionnaires, as items are not independent, i.e., changing the wording of one item might result in complex changes of the psychometric properties of other items. Therefore, iterative proceeding is recommended, conducting revisions with decreasing degrees of changes per block. Sixth, while no respondents reported too high cognitive load regarding the ranking of 7 items within a block, several respondents gave oral feedback that completing the negatively keyed blocks (ranking tasks 71 to 140) was more demanding than ranking positively keyed items (ranking tasks 1 to 70). Future studies should deal with the question of whether this effect is rather due to the items' keying or the sequence of the blocks. Finally, longitudinal analyses of MVSQ scores should be conducted in order to further explore temporal stability of individual value system preferences and predict behavioral criteria assumed to be affected by individual standing on value systems.

## Ethics statement

This study was carried out in accordance with recommendations of the Ethics Committee of the German Psychological Society (DGPS), with written informed consent obtained from all subjects. Full ethical review and approval was not required for this study in accordance with the national and institutional guidelines.

## Author contributions

TF developed the questionnaire, initiated the research project and collected data. JM conceived the study design, collected data and performed the data analysis. WS provided guidance and substantially contributed to the analysis and interpretation of data. JM wrote the first draft of the paper. WS and TF contributed to revisions. All authors approved the final version of the paper.

### Conflict of interest statement

The research being reported in this publication was supported by Develo GmbH by free access to perform the value system analysis (MVSQ/my motivation). The co-author TF is proprietor of Develo GmbH which is developing products like the ones related to the research being reported. The authors declare that the research was conducted in the absence of any commercial or financial relationships that could be construed as a potential conflict of interest.

## Footnotes

^1^For content descriptions of SVS value systems please see e.g., Schwartz (1994) or Bardi and Schwartz (2003).
